# No evidence for additional systemic eosinophil mobilization during exacerbations in patients with COPD and chronic bronchitis but no allergy

**DOI:** 10.3389/fmed.2025.1572291

**Published:** 2025-05-05

**Authors:** Anders Andersson, Kristina Andelid, Bettina Brundin, Ann Ekberg-Jansson, Anders Lindén

**Affiliations:** ^1^COPD Center, Department of Respiratory Medicine and Allergology, Sahlgrenska University Hospital, Gothenburg, Sweden; ^2^COPD Center, Department of Internal Medicine and Clinical Nutrition, Institute of Medicine, Sahlgrenska Academy, University of Gothenburg, Gothenburg, Sweden; ^3^Division for Lung and Airway Research, Institute of Environmental Medicine, Karolinska Institutet, Stockholm, Sweden; ^4^Närhälsan, Primary Care Administrative Management for Region Västra Götaland, Gothenburg, Sweden; ^5^Respiratory Medicine and Allergology, Department of Internal Medicine and Clinical Nutrition, Institute of Medicine, Sahlgrenska Academy, University of Gothenburg, Gothenburg, Sweden; ^6^Karolinska Severe COPD Center, Department of Respiratory Medicine and Allergy, and Center for Molecular Medicine, Karolinska University Hospital, Stockholm, Sweden

**Keywords:** biomarker, COPD, exacerbation, ECP, IL-4, systemic

## Abstract

**Background:**

There is published evidence that a modest increase in blood eosinophils during stable COPD indicates future risk for exacerbations and a potential utility of inhaled corticosteroids. This has been perceived as an argument for targeting systemic eosinophil mobilization to prevent exacerbations in COPD, but there are no published data on systemic eosinophil mobilization during exacerbations in patients without allergy.

**Methods:**

We investigated long-term tobacco smokers (LTS: ≥10 pack-years) with COPD and chronic bronchitis (COPD-CB; GOLD stage 1–4; *n* = 47) but no allergy; LTS without COPD and CB (LTS; *n* = 10), and healthy never-smokers (HNS; *n* = 10) during stable disease for cross-sectional comparisons. For longitudinal comparisons, we followed the COPD-CB group for 15 months during stable disease and exacerbations, excluding samples affected by systemic corticosteroids. We quantified blood concentrations of eosinophils, the activity marker eosinophilic cationic protein (ECP) and the chemokine interleukin (IL)-4.

**Results:**

During stable disease, the concentrations of eosinophils were similar for the COPD-CB and the LTS group, although higher than in the HNS group. The concentrations of ECP and IL-4 were not markedly different in the COPD-CB and LTS groups either. During exacerbations, the concentrations of eosinophils, ECP and IL-4 were not further increased, and there was even a mathematical trend towards a decrease for these concentrations.

**Conclusion:**

The clinical evidence presented here suggests that, by average, there is no additional mobilization of eosinophils during exacerbations in patients with COPD and chronic bronchitis but no allergy. Thus, in this common phenotype, the immunological rationale for targeting systemic eosinophils during exacerbations remains unaccounted for, which motivates verification studies in large cohorts stratified for allergy and chronic bronchitis.

## 1 Introduction

Chronic obstructive pulmonary disease (COPD) is the third most common cause of death at the global level, accounting for approximately 6 % of all deaths (1). Sadly, this cause of death can be expected to increase even more over time, given the global development of exposure to harmful agents like tobacco smoke, dust and particles, in combination with an aging population in many countries (1). Importantly, current pharmacotherapy in patients with COPD is not efficient enough for all clinical phenotypes and better tools for diagnosis and therapy are needed. To establish these, we need to improve the understanding of pathogenic mechanisms in distinct phenotypes of COPD.

During the last decade, it has been recognized that COPD may include several immunological endotypes, although there is limited understanding of how these relate to clinical phenotypes (1–5). However, it is known that the clinical phenotype of COPD with CB is signified by frequent exacerbations, associated with increased decline in lung function and poor long-term prognosis (1, 6–8). It is also known that exacerbations are especially common in the clinical phenotype with concomitant chronic bronchitis (9, 10). In addition, there is a growing body of evidence that the clinical phenotype with frequent exacerbations is associated with the endotype with enhanced blood concentrations of eosinophilic granulocytes (eosinophils) during stable disease, and there is published evidence that the concentrations of blood eosinophils mirror those of airway eosinophils (1, 11, 12). Interestingly, the endotype with these “enhanced eosinophil concentrations” responds favorably to inhaled corticosteroids (ICS) (1, 4, 13–19) and this has been perceived as an argument for targeting systemic eosinophil mobilization with medication to prevent exacerbations in COPD. However, most published studies on blood eosinophils in COPD have examined this biomarker during stable disease, and the present literature provides limited and conflicting information on systemic eosinophil mobilization during COPD exacerbations in patient populations of mixed phenotypes (13, 20). Despite several large cohort studies on blood eosinophils in COPD, there is only one recent study by Kang et al. that have addressed how the moderately enhanced systemic eosinophil mobilization in stable COPD relates to phenotypes having allergy, a comorbidity that is believed to affect a minority (20 to 25%) of the patients with COPD (21–23). Notably, the study by Kang et al. demonstrates that during stable disease, COPD patients with allergy have higher concentrations of blood eosinophils than those without allergy. Additionally, clinical trials of antibodies targeting eosinophil-mobilizing cytokine signaling indicate that inhibiting systemic eosinophil mobilization prevents from exacerbations in COPD, provided that the patients display blood concentrations of eosinophils above a critical threshold within the normal range or above (24, 25). Thus, there is clinical evidence compatible with modest systemic eosinophil mobilization not only being associated with but also mediating COPD exacerbations, but there is no conclusive information on systemic eosinophil mobilization per se during exacerbations in COPD patients with chronic bronchitis without allergy.

In the current study, we aimed to address whether systemic eosinophil mobilization is further enhanced during exacerbations in patients with COPD but no allergy. To address this, we investigated a population of well-characterized patients with mild to severe COPD. For these patients, current smoking, concomitant chronic bronchitis and a history of exacerbations constituted inclusion criteria, to increase the likelihood of exacerbations taking place during the course of our study. To avoid the confounding influence on eosinophil mobilization from asthma and allergy, both these comorbidities constituted exclusion criteria. We followed the included patients for 15 months, with sampling during stable disease and during exacerbations, for longitudinal within-subject (matched) comparisons. As control groups for cross-sectional comparisons, we investigated a group of healthy never-smokers (HNS) plus a group of long-term smokers without COPD and CB (LTS), applying asthma and allergy as exclusion criteria for these control groups as well. The subjects in the two control groups were investigated during the inclusion visit only. In all the study subjects, we assessed systemic eosinophil mobilization in three different ways, by quantifying blood concentrations of eosinophils, the eosinophil chemokine interleukin (IL)-4 and the eosinophil activity marker eosinophil cationic protein (ECP) (26, 27). Finally, we secured representative sputum samples at exacerbations to detect the putative presence of bacterial airway pathogens.

## 2 Methods

### 2.1 Ethics statement

The study was approved after review by the Regional Ethics Committee in Gothenburg (S 233–03, T 286–04, and T 521–06). The study subjects were included after oral and written informed consent, in accordance with the World Medical Association (the Helsinki Declaration). The complete protocol for the utilized study material has been published elsewhere and some data sets relating to clinical characteristics have been reported in other publications, although in a different scientific context (28–30).

### 2.2 Clinical protocol

In the COPD-CB group, we included long-term smokers (≥10 pack-years) with a diagnosis of COPD and CB, all without asthma and allergy. All these patients had a history of at least 1 exacerbation the last preceding year and they all displayed positive cotinine-test in urine (One-Step Cotinine Test^®^, UltiMed Products™, Ahrensburg, Germany) confirming their personal statements of current smoking (28, 29). Screening and inclusion were made during stable disease at the first visit. We then ascertained that there were no clinical signs of respiratory tract infection during the 4 preceding weeks, and no treatment with systemic corticosteroids within this time frame either. During this first visit, the study physician secured a negative history of allergy and, whenever in doubt, secured a blood test for specific IgE against common allergens (Phadiatop™, Phadia, Uppsala, Sweden). Only subjects with a negative specific IgE test were included in the study (3 in the COPD-CB group and 3 in the control groups). All the patients in the COPD-CB group underwent a physical examination and dynamic spirometry plus test of gas diffusion capacity (DLCO). Because all these patients had a spirometry-verified prior COPD diagnosis prior to the first study visit, the functional tests during the first visit were performed without additional bronchodilation. In addition, these patients also underwent a regular pulmonary X-ray to exclude additional pulmonary comorbidities. After the first visit, the patients in the COPD-CB group were monitored and examined during stable disease every 15th week during a total period of 60 weeks (2–5 visits). In the case of a clinical exacerbation, the patient was examined during an extra visit when study sampling was ascertained during daytime office hours prior to emergency treatment. Signs of infection in the lungs were deemed as exacerbation of COPD. During all regular visits and during exacerbations, we collected venous blood samples as specified below. We interviewed all patients to ascertain that none of the patients delivering samples had been treated with systemic (oral or intravenous) corticosteroids within 4 weeks prior to sampling. All patients in the COPD-CB group were instructed to continue their regular medication throughout the course of the entire study. As control groups, we included HNS and LTS who all lacked allergy and asthma, examined during a first visit in the same way as the patients in the COPD-CB group. For inclusion, the subjects in the two control groups had to display a normal lung function, including both ventilatory volumes and gas diffusion capacity, without history or signs of CB, dyspnea or phlegm.

### 2.3 Blood samples

Blood samples from all study subjects underwent analysis of automated blood cell differential counts (ADVIA 2120i Hematology system^®^; Siemens, Duisburg, Germany) at the Department of Clinical Chemistry at Sahlgrenska University Hospital, Gothenburg, Sweden, to determine concentrations of granulocytes. For all study subjects, we also analyzed fresh plasma samples for concentration of C-reactive protein using a high-sensitivity assay (HS-CRP, Tina–quant C-reactive protein high-sensitivity assay^®^, No 1972944001; Hoffmann-La Roche Ltd.^®^, Basel, Switzerland) in accordance with the manufacturer’s instructions at the Department of Clinical Chemistry, Sahlgrenska University Hospital. For the analysis of markers for recruitment and activation of eosinophils, we utilized frozen (-80°C) plasma samples. In these samples, we quantified the concentrations of ECP and IL-4 using commercial ELISA kits (ECP ELISA Kit, EIAab™, Wuhan, Peoples Republic of China and Human IL-4, R&D Systems™, Minneapolis, MN, United States). For ECP only, some values exceeded the highest value of the standard curve, and these values were assigned this maximum value as an arbitrary assessment (ie. 360 ng/mL). For IL-4 only, some samples generated values below the lowest concentration of the standard curve (0.64 pg./mL). These samples were assigned the mean value of zero and the lowest and approved value of the standard curve as an arbitrary assessment (i.e., 0.32 pg./mL). Due to depletion of samples from the original study material, some of these analyses could not be made for all subjects, as indicated in the results section.

### 2.4 Sputum samples

We collected sputum samples during exacerbations as previously described (29). We only included data from sputum samples deemed likely or definitively to be representative for the lower airways in terms of containing less than 1% squamous epithelial cells (31). During exacerbation, 18 sputum samples were collected from different subjects (from their first exacerbation). The data on bacterial growth was divided in two principal groups—bacteria representing normal oropharyngeal flora (Normal flora) and bacterial pathogens (Pathogens), respectively. If there was growth of both principal groups, the sample was classified as having growth of pathogens. The two groups were compared with respect to concentrations of eosinophils, IL-4 and ECP. In three subjects, IL-4 and ECP were analyzed in samples from the second exacerbation, due to depletion of exacerbation samples from the first one.

### 2.5 Statistical methods

For this explorative study, we utilized a human study material that has previously proved to possess the statistical power needed to demonstrate established group differences for patients with COPD and chronic bronchitis versus controls (28–30). For the current study, non-parametric statistics were applied and, in general, data are presented both as individual and median values (range). All analyses were performed using the software IBM SPSS Statistics Version 25 (Chicago, Illinois, United States). *p* < 0.05 was regarded as statistically significant.

## 3 Results

### 3.1 Clinical material

**Figure 1:**
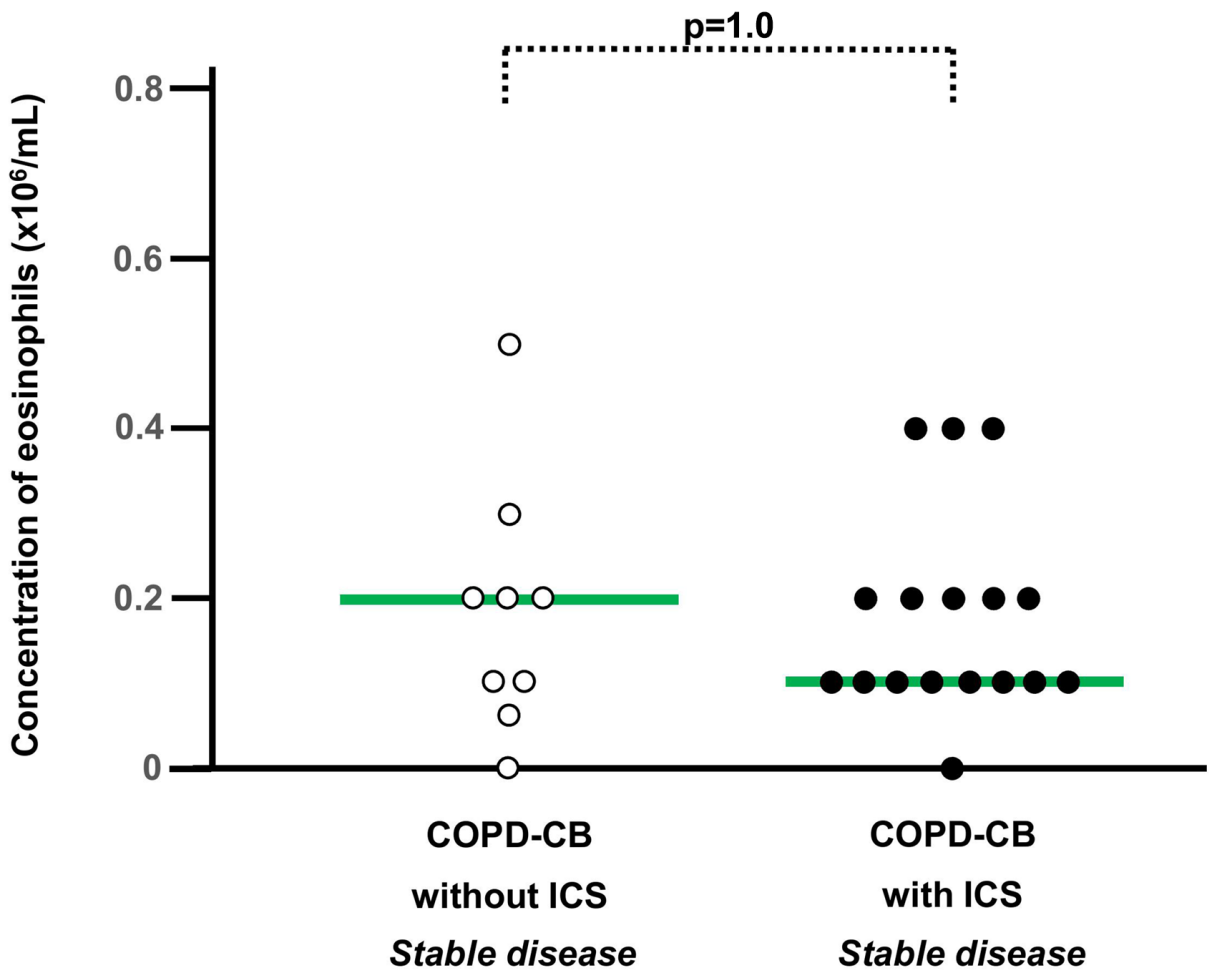
The blood concentrations of eosinophils during stable disease in 26 long-term smokers with exacerbations of COPD and chronic bronchitis (COPD-CB) with and without treatment with inhaled corticosteroids (ICS). The data are presented as individual values with medians (bold lines). The *p*-value was determined using Wilcoxon signed rank test.

We included samples during stable disease from 47 patients in the COPD-CB group, out of which 26 patients delivered exacerbation samples at the study center (Table 1). Importantly, none of the patients delivering samples had been treated with systemic (oral or intravenous) corticosteroids within 4 weeks prior to sampling. For the control groups, we included samples from 10 subjects in the HNS and from 10 subjects in the LTS group (Table 1). In terms of clinical characteristics, the range for age was of a similar order of magnitude and overlapped for all study groups (Table 1). Moreover, the range for tobacco load (i.e., number of pack-years) was of a similar order of magnitude for the study groups including smokers. The lung function data displayed the expected characteristics in the COPD-CB group (Table 1), whereas lung function was normal or close to normal in the two control groups. Seventeen of the 26 patients with exacerbations in the COPD-CB group utilized ICS as part of their regular treatment, without any statistically significant difference in blood concentrations of eosinophils during stable disease (Figure 1). However, in the COPD-CB group, the subgroups with and without exacerbations displayed enhanced blood concentrations of CRP during stable disease (Table 1). The blood concentrations of neutrophils tended to be higher in the COPD-CB group during stable disease and in the LTS group, compared with the HNS group.

**Table 1:** Demographic characteristics during stable disease for 10 healthy never-smokers (HNS), 10 current long-term smokers (LTS), 47 current long-term smokers with COPD and chronic bronchitis (COPD-CB) including 26 patients with exacerbations and 21 patients with no such exacerbation (COPD-CB). There was no treatment with oral glucocorticoids within 4 weeks prior to sampling.

	HNS	LTS	COPD-CB	COPD-CB
	All samples		Exacerbation samples	No exacerbation samples
Number (n)	10	10	26	21
Age (years)	68 (47–70)	50 (25–64)	61 (46–74)	65 (46–76)
Male/female	3/7	2/8	9/17	9/12
Pack-years	0	28 (14–42)	32 (14–80)	44 (10–104)
FEV1 (L)	2.8 (1.9–3.7)	3.0 (2.3–4.1)	1.6 (0.7–2.6)	1.8 (0.8–2.8)
FEV1 (% of pred.)	120 (97–137)	106 (83–119)	57 (29–86)	67 (41–97)
FEV1/FVC	79 (73–84)	77 (73–84)	56 (29–69)	57 (36–68)
DLCO (% of pred.)	98 (75–139)	87 (77–111)	68 (44–100)	72 (47–101)
CRP (mg/L)	0.8 (0.2–5.7)	0.7 (O.2–5.8)	2.3 (0.3–15)	1.7 (0.5–8)
Neutrophils (x10^9^/L)	2.8 (1.8–4.7)	4.4 (2.5–7.8)	4.1 (2.3–9.6)	5.4 (2–9.1)

### 3.2 Eosinophil mobilization during stable disease

**Figure 2:**
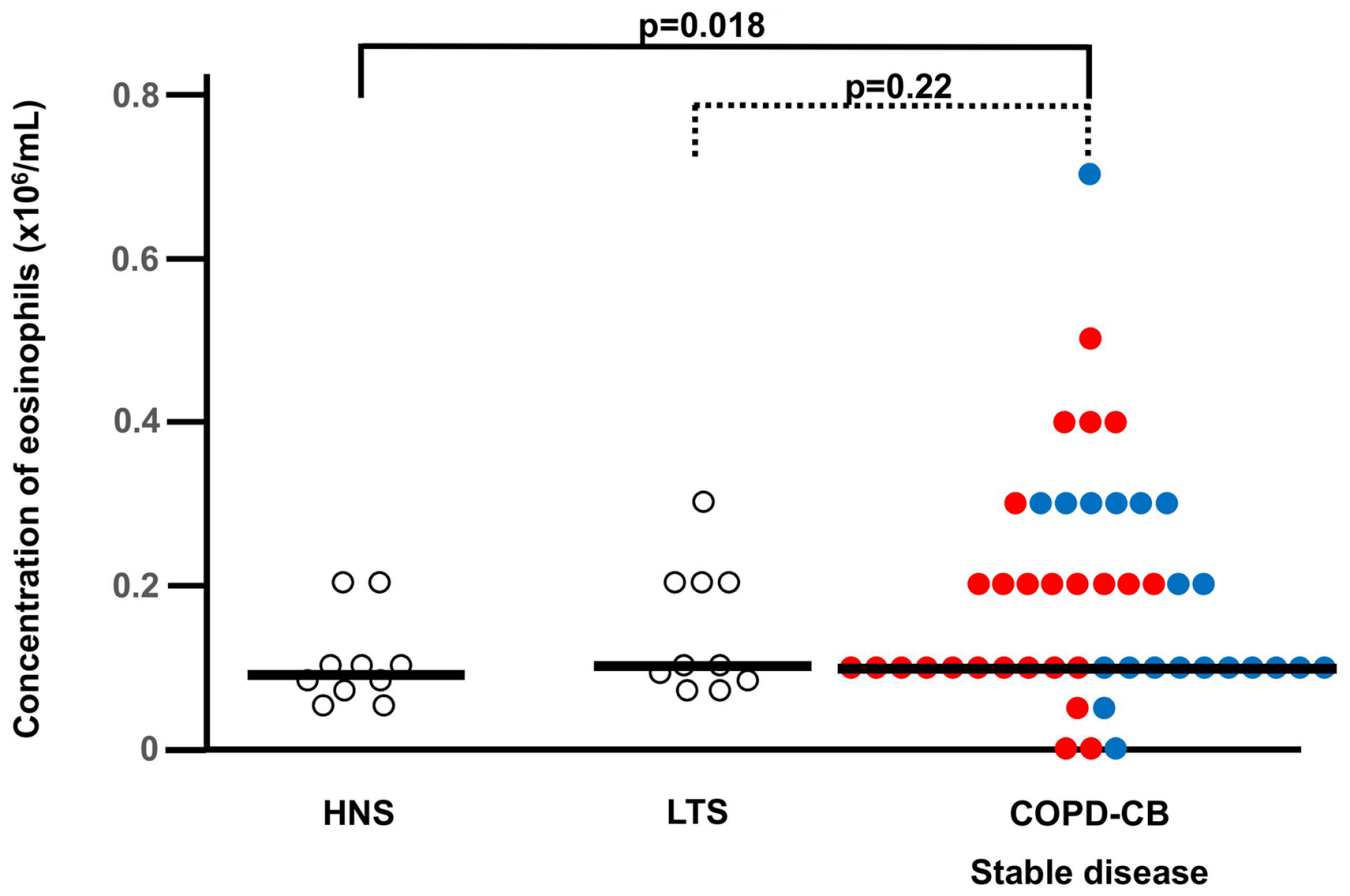
The blood concentrations of eosinophils during stable disease in current long-term smokers with COPD and chronic bronchitis (COPD-CB) in comparison with healthy never-smokers (HNS) and current long-term tobacco smokers without COPD and chronic bronchitis (LTS). Patients in the COPD-CB group with exacerbations of COPD are represented by red dots and those with no exacerbations of COPD are represented by blue dots. The concentrations of eosinophils are presented as individual values with medians (bold lines). The *p*-value was determined using Kruskal-Wallis test followed by Mann–Whitney U-test.

**Figure 3:**
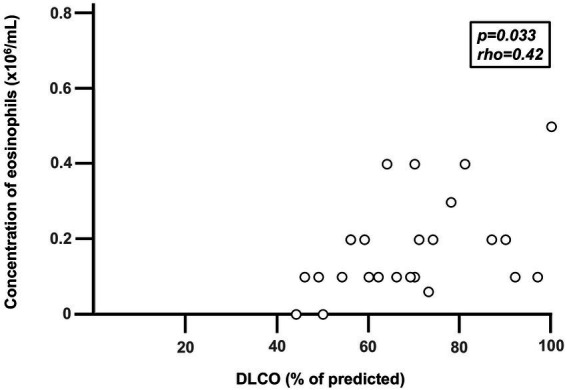
Correlation between diffusion capacity for carbon dioxide (DLCO) and the blood concentration of eosinophils during stable disease in 26 long-term smokers with exacerbations of COPD and chronic bronchitis (COPD-CB). Data are presented as individual values. Three subjects display the same value (overlap) in the figure. The *p*- and *r*-values were determined using Spearman Rank Correlation test.

During stable disease in the COPD-CB group, the blood concentrations of eosinophils were higher than in the HNS group, while the COPD-CB group and the LTS group displayed similar concentrations (Figure 2). More specifically, 12 out of 47 patients (26%) with COPD and CB displayed an eosinophil concentration of 0.3 × 10^6^/mL or higher (Figure 2). Notably, there was no statistically significant correlation between the blood concentrations of eosinophils during stable disease and the number of (future) exacerbations in the COPD-CB group during the study (*p* = 0.14; rho = 0.30; *n* = 26; Spearman Rank Correlation test, data not shown). Nor did patients with two or more future exacerbations display any statistically significant difference in blood concentrations of eosinophils during stable disease in comparison with those who had one future exacerbation only (0.2 vs. 0.1 × 10^6^ cells/L; *p* = 0.24; *n* = 26; Mann–Whitney U-test, data not shown). During stable disease in the COPD-CB group, the concentrations of ECP tended to be somewhat higher and IL-4 tended to be lower than the control groups, even if these changes were not statistically significant (Table 2). For patients in the COPD-CB group who were treated with ICS and delivered exacerbation samples, there was a trend toward a lower eosinophil concentration during stable disease, although without statistical significance (Figure 1). During stable disease in the COPD-CB group, we detected no correlations between concentrations of eosinophils, ECP or IL-4 in blood, on the one hand, and FEV_1_ (% predicted) or the FEV_1_/FVC ratio, on the other hand (Supplementary Figures 1A,B, 2A,B, 3A,B). Under these conditions, however, we did detect a positive correlation between the concentration of eosinophils in blood and gas diffusion capacity (DLCO % of predicted; Figure 3). No matching correlations were detected for either concentrations of ECP or IL-4 in blood under these conditions (Supplementary Figures 2C, 3C). We failed to detect any correlation between the concentration of eosinophils and partial pressure of oxygen in the COPD-CB group during stable disease (Supplementary Figure 4).

**Table 2:** The concentrations of eosinophil cationic protein (ECP) and interleukin-4 (IL-4) in blood during stable clinical conditions for 10 (*n* = 9 for ECP) healthy never-smokers (HNS), 10 current long-term smokers (LTS) and 26 (*n* = 25 for IL-4) current long-term smokers with exacerbations of COPD and chronic bronchitis (COPD-CB).

	HNS	LTS	COPD-CB
ECP ng/ml	33.3 (6.5–360)	26.5 (7.3–323.2)	67.6 (1.6–360)
IL-4 pg/mL	13.7 (0.3–628.4)	16.4 (0.3–969.2)	1.7 (0.3–91.2)

No statistically significant group differences were detected. The *p*-value was determined using Kruskal-Wallis test (*p* = 0.27 and *p* = 0.17, respectively).

### 3.3 Eosinophil mobilization during exacerbations

**Figure 4:**
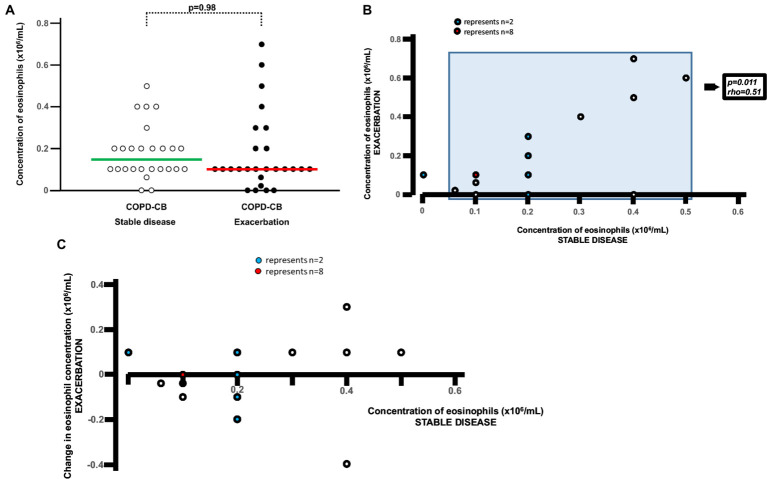
Eosinophils in blood during stable disease and during exacerbations in long-term smokers with exacerbations of COPD and chronic bronchitis. **(A)** Absolute blood concentrations for eosinophils presented as individual values with medians (bold lines) during stable disease and exacerbation. The p-value was determined using Wilcoxon signed rank test. **(B)** Correlation between absolute blood concentration of eosinophils during stable disease and during exacerbation. The *p*- and *r*-values were determined for patients with eosinophil concentrations above zero, using Spearman Rank Correlation test. **(C)** Individual change in blood concentration of eosinophils during exacerbation versus absolute blood concentration during stable disease for long-term tobacco smokers with COPD and chronic bronchitis for each patient during stable disease.

**Figure 5:**
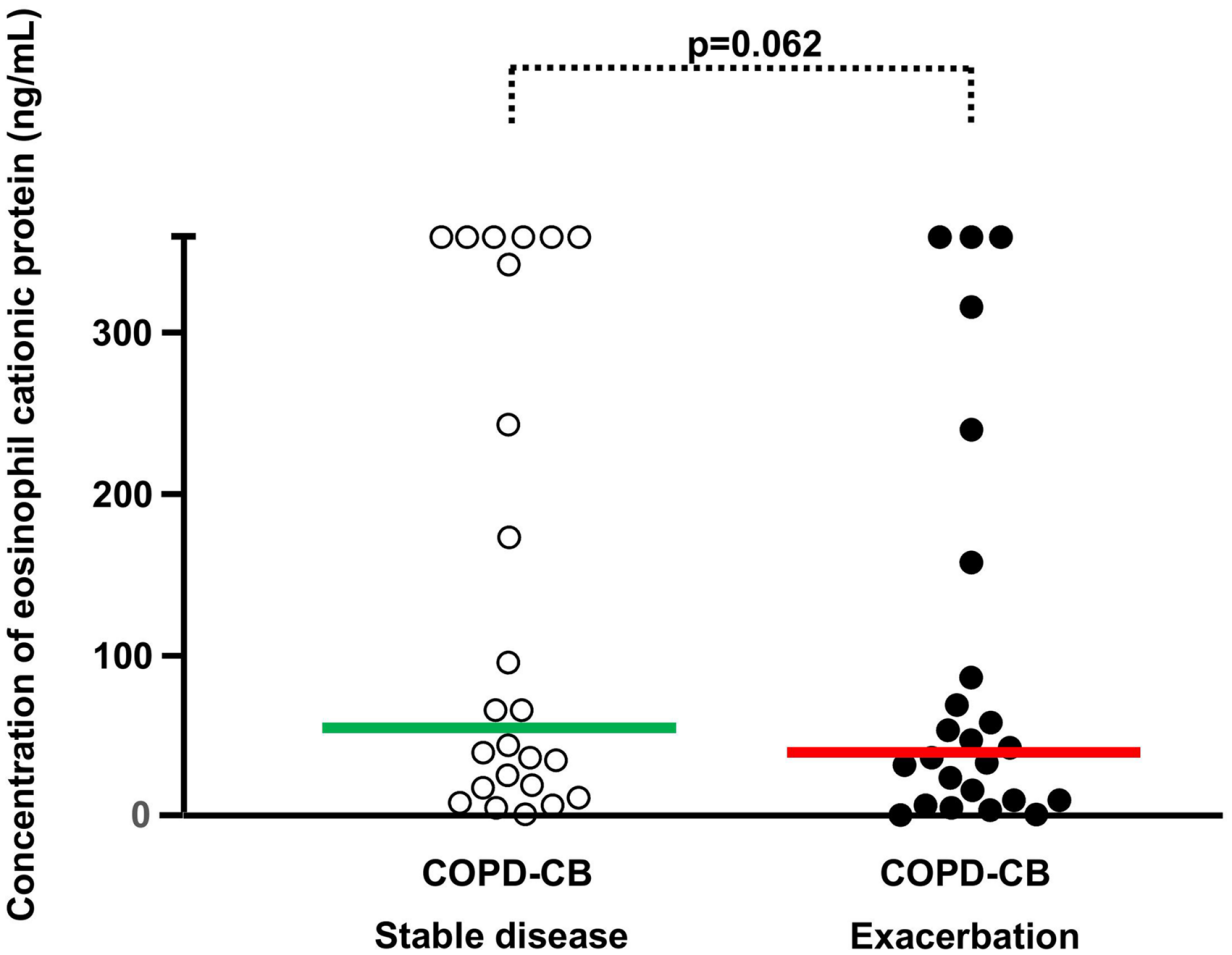
The blood concentrations of eosinophilic cationic protein (ECP) during stable disease and exacerbations in 24 current long-term smokers with exacerbations of COPD and chronic bronchitis (COPD-CB). The data are presented as individual values with medians (bold lines). The *p*-value was determined using Wilcoxon signed rank test.

**Figure 6:**
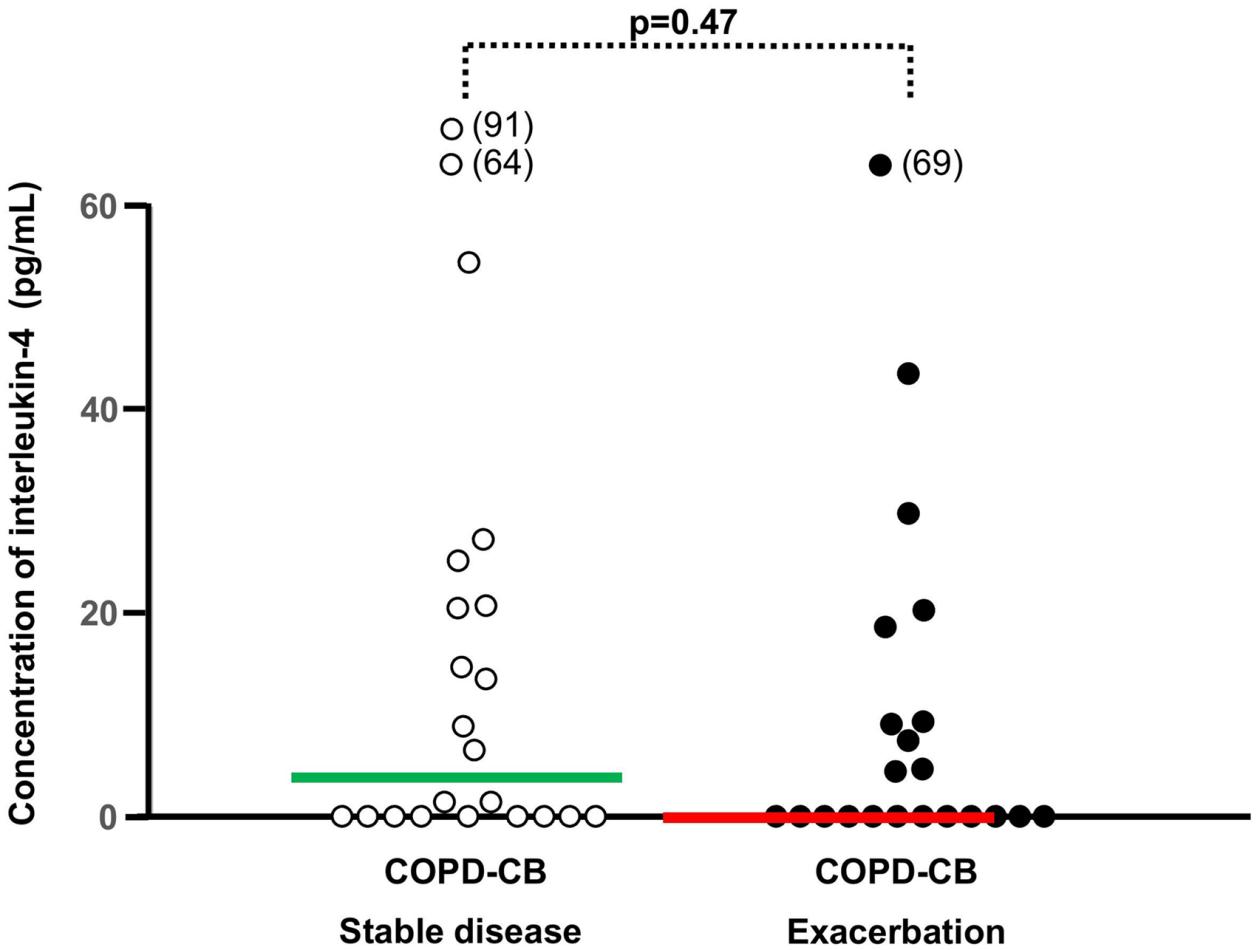
The blood concentrations of interleukin (IL)-4 during stable disease and exacerbations in 22 current long-term smokers with exacerbations of COPD and chronic bronchitis (COPD-CB). The data are presented as individual values with medians (bold lines). The p-value was determined using Wilcoxon signed rank test.

During exacerbations in the COPD-CB group, there was no additional increase in the concentration of eosinophils in blood (Figure 4A). On the contrary, there was a mathematical trend toward a decrease in these concentrations, although this trend was not statistically significant. When addressing the association of the eosinophil concentration during stable disease with that during exacerbations (Figure 4B), we did detect a positive and statistically significant correlation. However, we found no evidence that the change in eosinophil concentration during exacerbation did relate to the eosinophil concentration during stable disease (Figure 4C). At the individual level, we observed qualitatively different alterations of the eosinophil concentration in blood, with 5 patients displaying an increase of 50% or more and 7 patients displaying a decrease of 50% or more (Figure 4C). During exacerbations in the COPD-CB group, there was no additional increase in the concentrations of either ECP or IL-4 in blood (Figures 5, 6). On the contrary, there was a mathematical trend toward a decrease in each of these concentrations, although neither of these trends proved to be statistically significant.

### 3.4 Bacterial growth

During exacerbations, no corresponding difference was observed for either the concentration of eosinophils, ECP or that of IL-4 when comparing normal flora and pathogens (Supplementary Table 1).

## 4 Discussion

Given the current interest in the eosinophil as a biomarker and potential therapeutic target in COPD, we find it important to address whether there is an additional increase in systemic eosinophil mobilization during exacerbations in COPD patients with chronic bronchitis but no allergy (5, 19, 21, 24). This is because, as addressed above, chronic bronchitis is a very important clinical phenotype at risk for frequent exacerbations whereas allergy affects only a minority of COPD patients (1, 6–10, 22, 23), but also because of the fact that during stable disease, patients with COPD and allergy have clearly higher eosinophil concentrations in blood compared to those without allergy (21). Along these lines, we specifically targeted the blood concentrations of eosinophilic granulocytes per se, the activity marker ECP and the chemokine IL-4 in blood in carefully characterized patients with COPD and chronic bronchitis but no allergy. In these patients, we found that neither of the three “eosinophilic outcomes” were further enhanced during exacerbation. This was the case even though we facilitated detection of alterations by using the matched samples from stable disease and exacerbations within each patient. In fact, we detected trends in the opposite direction, observations providing an even stronger argument against additional mobilization of eosinophils in this clinical setting. Notably, during stable disease, a small portion (26%) of the included patients with COPD and CB displayed eosinophil concentrations that are known to associate with significant risk for exacerbations, in line with previous studies (14, 16). Moreover, we obtained no evidence that the exacerbation-related alteration in the concentration of eosinophils related to the concentration during stable disease although we did verify a correlation of high eosinophil concentrations during exacerbation with high corresponding concentrations during stable disease (5, 21). To us, it seems likely that these results relate to the fact that we excluded COPD patients with the Type 2-driven comorbidity allergy from our study.

Although the existing literature is very limited in this respect, it does contain support for our main finding that there are no signs of further increase in systemic eosinophil mobilization during exacerbations in patients with COPD and chronic bronchitis but no allergy. This support consists of one previous publication reporting a study that did contain evidence for a trend toward a decrease in the concentration of eosinophils in blood during exacerbations of COPD (20). This makes it likely that the lack of additional systemic eosinophil mobilization is representative of exacerbations of COPD per se, at least for the phenotype with current smoking and chronic bronchitis but no allergy or asthma. Clearly, this contrasts to what has been observed in patients with exacerbations of asthma (32). In the literature, there is one study indicating that local mobilization of eosinophils may occur during exacerbations of patients with chronic bronchitis (33). In that pioneering study, four out of 11 patients with exacerbations and six out of 12 with stable disease displayed an FEV_1_/VC ratio suggesting airflow obstruction but no information on reversibility, making it uncertain whether these patients suffered from chronic or reversible airflow obstruction, in addition to chronic bronchitis. Moreover, the patients were claimed to be “non-atopic,” seemingly based upon a lack of history for allergic rhinitis, whereas the studied patients were heterogeneous with reference to current and former smoking. Local eosinophil mobilization was assessed as the number of these granulocyte subsets in cell differential counts performed either in induced sputum or bronchial tissue samples, as well as quantitative staining for EG-2 in bronchial tissue samples. Interestingly, all these assessments of local eosinophil mobilization displayed more signal among patients with exacerbations compared with those who had stable disease, although the observations were not paired or matched. In view of the referred study, we think that it is relevant to point out that our current study does not include any information on eosinophil mobilization in the airways, and thus we cannot rule out such local mobilization during COPD exacerbations.

Importantly, during the emergency visits for the patients in the COPD-CB group, we consistently harvested our exacerbation samples from patients prior to the standardized exacerbation treatment with oral corticosteroids and oral antibiotics. In doing so, we avoided the confounding influence of systemic and local pharmacotherapy. This means that we can rule out that either of these clinical drug treatments accounted for the lack of an increase in the concentrations of eosinophils, ECP and IL-4 during exacerbations. Moreover, during stable disease, we detected no statistically significant difference in the markers of eosinophil mobilization relating to whether the patients utilized inhaled corticosteroids or not, and the same was true for blood concentrations of eosinophils during exacerbations. This outcome is supported by previous studies suggesting that the impact of inhaled corticosteroids on inflammatory outcomes is modest in patients with COPD and chronic bronchitis, although we cannot rule out that limitations in statistical power may have confounded our current study (1, 3, 10).

Notably, there are several arguments in favor of our study material being representative for COPD among current smokers even if the study population is of a modest size. Both sexes were represented, all patients had a substantial historic tobacco load of the same order of magnitude, relatively high age, slightly enhanced CRP and neutrophil concentrations in blood, and they utilized inhalation therapy according to relevant guidelines, including ICS in 65 % of the study population. The same argument applies to the fact that the blood concentrations of eosinophils were modestly enhanced in the COPD-CB group during stable disease, compared with what was the case in the healthy never-smokers, fully compatible with what has previously been reported (16, 34). Moreover, we observed a trend only toward a difference in the blood concentrations of eosinophils during stable disease for patients with and without ICS, compatible with a limited effect of this therapy on systemic eosinophil mobilizations in COPD. Furthermore, as expected, some patients in our study population did display signs of local growth of bacterial pathogens during exacerbations. However, we did not detect any statistically significant difference in blood concentrations of either eosinophils, ECP or IL-4 with and without growth of these pathogens, a finding that is compatible with bacterial stimulation not being an important determinant for eosinophil mobilization in exacerbations of COPD without allergy. We think that at least a trend toward increase would have been detectable if a strong association does exist in the phenotype that we examined. For factual reasons, we find it hard to believe that the absence of an average increase that support systemic eosinophil mobilization in COPD exacerbations in our study would be due to limitations in statistical power only. This is because our material has previously displayed sufficient statistical power to demonstrate statistically significant group differences and correlations for a range of clinical and immunological outcomes (28–30). Examples of this include CRP and immune cells such as lymphocytes and neutrophils, cytokines such as IL-17A and GRO, plus effector molecules such as neutrophil elastase, myeloperoxidase and galectin-3 (28–30). In addition to this, we did detect a trend toward a decrease for three different eosinophil-related outcomes during exacerbations. Given the fact that Kang et al. have established that allergy is associated with enhanced systemic eosinophil concentrations in patients with stable COPD (21), we think that our consistent exclusion of patients and control subjects with clinical manifestations of allergy was the decisive factor explaining the negative outcome in terms of systemic eosinophil mobilization in our current study. In this context, we find it relevant to point out that the investigators responsible for previous studies on systemic eosinophil mobilization in COPD have been either inconsistent in or omitted information about their handling of the comorbidity allergy (13, 14, 16, 18, 19, 24). The latter implies that patients classified as “COPD” in previous studies may in fact have been suffering from the comorbidity allergy, and in some studies from asthma as well, with an expected impact on Type 2-driven immunological events including enhanced systemic eosinophil mobilization. Along these lines, a very recent study on an antibody that blocks IL-4 and IL-13 (dupilumab) in patient with COPD but no concomitant asthma demonstrated positive effects on lung function as well as a reduction in exacerbations, in a patient population selected based upon a modest enhancement in blood concentrations of eosinophils (≥0.3 × 10^6^ cells/mL), compatible with enhanced Type 2-signaling (24). To us, this is a strong argument for either consistently stratifying patients in or focusing on a well-defined clinical phenotype in future studies of systemic as well as local eosinophil mobilization in the airways in patients with COPD.

## 5 Conclusion

In conclusion, our study on a carefully characterized cohort of patients with COPD and chronic bronchitis but no allergy forwards clinical evidence that, by average, there is no additional, systemic eosinophil mobilization during exacerbations in the clinical phenotype that was examined. Thus, in this common and important phenotype, the immunological rationale for targeting systemic eosinophils during exacerbations remains unaccounted for. We think that this key finding motivates verifications studies on systemic and local eosinophil mobilization during exacerbations in large cohorts of COPD patients stratified for both chronic bronchitis and allergy.

## Data Availability

The raw data supporting the conclusions of this article will be made available by the authors, by request.

## References

[1] GOLD is an world wide organization behind this document and publish yearly an updated consensus document about the diagnosis and management of COPD. Global Initiative for Chronic Obstructive Lung Disease. (2025).

[2] Guerreiro I Soccal PM. Les phénotypes de la BPCO. Rev Med Suisse. (2019) 15:2082–6. doi: 10.53738/REVMED.2019.15.671.2082 31742938

[3] Corlateanu A Mendez Y Wang Y Garnica RJA Botnaru V Siafakas N. Chronic obstructive pulmonary disease and phenotypes: a state-of-the-art. Pulmonology. (2020) 26:95–100. doi: 10.1016/j.pulmoe.2019.10.006, PMID: 31740261

[4] David B Bafadhel M Koenderman L De Soyza A. Eosinophilic inflammation in COPD: from an inflammatory marker to a treatable trait. Thorax. (2021) 76:188–95. doi: 10.1136/thoraxjnl-2020-215167, PMID: 33122447 PMC7815887

[5] Mayhew D Devos N Lambert C Brown JR Clarke SC Kim VL. Longitudinal profiling of the lung microbiome in the AERIS study demonstrates repeatability of bacterial and eosinophilic COPD exacerbations. Thorax. (2018) 73:422–30. doi: 10.1136/thoraxjnl-2017-210408, PMID: 29386298 PMC5909767

[6] Hillas G Perlikos F Tzanakis N. Acute exacerbation of COPD: is it the "stroke of the lungs"? Int J Chron Obstruct Pulmon Dis. (2016) 11:1579–86. doi: 10.2147/COPD.S106160, PMID: 27471380 PMC4948693

[7] Oussedik F Khelafi R Skander F. Impact des exacerbations aigües de BPCO sur la mortalité. Rev Mal Respir. (2019) 36:7–14. doi: 10.1016/j.rmr.2017.12.005, PMID: 30612748

[8] Kim V Criner GJ. Chronic bronchitis and chronic obstructive pulmonary disease. Am J Respir Crit Care Med. (2013) 187:228–37. doi: 10.1164/rccm.201210-1843CI, PMID: 23204254 PMC4951627

[9] Kim V Han MK Vance GB Make BJ Newell JD Hokanson JE. The chronic bronchitic phenotype of COPD: an analysis of the COPDGene study. Chest. (2011) 140:626–33. doi: 10.1378/chest.10-2948, PMID: 21474571 PMC3168856

[10] Kim V Zhao H Boriek AM Anzueto A Soler X Bhatt SP. Persistent and newly developed chronic bronchitis are associated with worse outcomes in chronic obstructive pulmonary disease. Ann Am Thorac Soc. (2016) 13:1016–25. doi: 10.1513/AnnalsATS.201512-800OC, PMID: 27158740 PMC5015750

[11] Maetani T Tanabe N Sato A Shiraishi Y Sakamoto R Ogawa E. Association between blood eosinophil count and small airway eosinophils in smokers with and without COPD. ERJ Open Res. (2023) 9:00235–2023. doi: 10.1183/23120541.00235-2023, PMID: 37868149 PMC10588801

[12] Barnes PJ. Inflammatory endotypes in COPD. Allergy. (2019) 74:1249–56. doi: 10.1111/all.13760, PMID: 30834543

[13] Bafadhel M McKenna S Terry S Mistry V Reid C Haldar P. Acute exacerbations of chronic obstructive pulmonary disease: identification of biologic clusters and their biomarkers. Am J Respir Crit Care Med. (2011) 184:662–71. doi: 10.1164/rccm.201104-0597OC, PMID: 21680942

[14] Vedel-Krogh S Nielsen SF Lange P Vestbo J Nordestgaard BG. Blood eosinophils and exacerbations in chronic obstructive pulmonary disease. The Copenhagen general population study. Am J Respir Crit Care Med. (2016) 193:965–74. doi: 10.1164/rccm.201509-1869OC, PMID: 26641631

[15] Bafadhel M Pavord ID Russell REK. Eosinophils in COPD: just another biomarker? Lancet Respir Med. (2017) 5:747–59. doi: 10.1016/S2213-2600(17)30217-5, PMID: 28601554

[16] Pascoe S Barnes N Brusselle G Compton C Criner GJ Dransfield MT. Blood eosinophils and treatment response with triple and dual combination therapy in chronic obstructive pulmonary disease: analysis of the IMPACT trial. Lancet Respir Med. (2019) 7:745–56. doi: 10.1016/S2213-2600(19)30190-0, PMID: 31281061

[17] Mac Leod M Papi A Contoli M Beghe B Celli BR Wedzicha JA. Chronic obstructive pulmonary disease exacerbation fundamentals: diagnosis, treatment, prevention and disease impact. Respirology. (2021) 26:532–51. doi: 10.1111/resp.14041, PMID: 33893708

[18] Li J Liang L Feng L Cao S Cai YS Li X. The prognostic value of blood eosinophil level in AECOPD is influenced by corticosteroid treatment during hospitalization. J Inflamm Res. (2023) 16:3233–43. doi: 10.2147/JIR.S421605, PMID: 37555013 PMC10404713

[19] Singh D Criner GJ Agusti A Bafadhel M Soderstrom J Luporini Saraiva G. Benralizumab prevents recurrent exacerbations in patients with chronic obstructive pulmonary disease: a post hoc analysis. Int J Chron Obstruct Pulmon Dis. (2023) 18:1595–9. doi: 10.2147/COPD.S418944, PMID: 37533773 PMC10390712

[20] Schumann DM Tamm M Kostikas K Stolz D. Stability of the blood eosinophilic phenotype in stable and exacerbated COPD. Chest. (2019) 156:456–65. doi: 10.1016/j.chest.2019.04.012, PMID: 31047957

[21] Kang HS Kim SK Kim YH Kim JW Lee SH Yoon HK. The association between eosinophilic exacerbation and eosinophilic levels in stable COPD. BMC Pulm Med. (2021) 21:74. doi: 10.1186/s12890-021-01443-4, PMID: 33653314 PMC7923497

[22] Jamieson DB Matsui EC Belli A McCormack MC Peng E Pierre-Louis S. Effects of allergic phenotype on respiratory symptoms and exacerbations in patients with chronic obstructive pulmonary disease. Am J Respir Crit Care Med. (2013) 188:187–92. doi: 10.1164/rccm.201211-2103OC, PMID: 23668455 PMC3778754

[23] Fattahi F ten Hacken NH Lofdahl CG Hylkema MN Timens W Postma DS. Atopy is a risk factor for respiratory symptoms in COPD patients: results from the EUROSCOP study. Respir Res. (2013) 14:10. doi: 10.1186/1465-9921-14-10, PMID: 23356508 PMC3599617

[24] Bhatt SP Rabe KF Hanania NA Vogelmeier CF Bafadhel M Christenson SA. Dupilumab for COPD with blood eosinophil evidence of type 2 inflammation. N Engl J Med. (2024) 390:2274–83. doi: 10.1056/NEJMoa2401304, PMID: 38767614

[25] Fitz Gerald JM Bleecker ER Nair P Korn S Ohta K Lommatzsch M. Benralizumab, an anti-interleukin-5 receptor alpha monoclonal antibody, as add-on treatment for patients with severe, uncontrolled, eosinophilic asthma (CALIMA): a randomised, double-blind, placebo-controlled phase 3 trial. Lancet. (2016) 388:2128–41. doi: 10.1016/S0140-6736(16)31322-8, PMID: 27609406

[26] Pritam P Manna S Sahu A Swain SS Ramchandani S Bissoyi S. Eosinophil: a central player in modulating pathological complexity in asthma. Allergol Immunopathol (Madr). (2021) 49:191–207. doi: 10.15586/aei.v49i2.50, PMID: 33641309

[27] Rosenberg HF Phipps S Foster PS. Eosinophil trafficking in allergy and asthma. J Allergy Clin Immunol. (2007) 119:1303–10. doi: 10.1016/j.jaci.2007.03.048, PMID: 17481712

[28] Andelid K Tengvall S Andersson A Levanen B Christenson K Jirholt P. Systemic cytokine signaling via IL-17 in smokers with obstructive pulmonary disease: a link to bacterial colonization? Int J Chron Obstruct Pulmon Dis. (2015) 10:689–702. doi: 10.2147/COPD.S76273, PMID: 25848245 PMC4381892

[29] Andelid K Andersson A Yoshihara S Ahren C Jirholt P Ekberg-Jansson A. Systemic signs of neutrophil mobilization during clinically stable periods and during exacerbations in smokers with obstructive pulmonary disease. Int J Chron Obstruct Pulmon Dis. (2015) 10:1253–63. doi: 10.2147/COPD.S77274, PMID: 26170654 PMC4493974

[30] Sundqvist M Andelid K Ekberg-Jansson A Bylund J Karlsson-Bengtsson A Linden A. Systemic Galectin-3 in smokers with chronic obstructive pulmonary disease and chronic bronchitis: the impact of exacerbations. Int J Chron Obstruct Pulmon Dis. (2021) 16:367–77. doi: 10.2147/COPD.S283372, PMID: 33642857 PMC7903965

[31] Qvarfordt I Riise GC Andersson BA Larsson S. Lower airway bacterial colonization in asymptomatic smokers and smokers with chronic bronchitis and recurrent exacerbations. Respir Med. (2000) 94:881–7. doi: 10.1053/rmed.2000.0857, PMID: 11001080

[32] Nakagome K Nagata M. Involvement and possible role of eosinophils in asthma exacerbation. Front Immunol. (2018) 9:2220. doi: 10.3389/fimmu.2018.02220, PMID: 30323811 PMC6172316

[33] Saetta M Di Stefano A Maestrelli P Turato G Ruggieri MP Roggeri A. Airway eosinophilia in chronic bronchitis during exacerbations. Am J Respir Crit Care Med. (1994) 150:1646–52. doi: 10.1164/ajrccm.150.6.7952628, PMID: 7952628

[34] Roche N Chapman KR Vogelmeier CF Herth FJF Thach C Fogel R. Blood eosinophils and response to maintenance chronic obstructive pulmonary disease treatment. Data from the FLAME trial. Am J Respir Crit Care Med. (2017) 195:1189–97. doi: 10.1164/rccm.201701-0193OC, PMID: 28278391

