# Supplementary figures and images for: No evidence for additional systemic eosinophil mobilization during exacerbations in patients with COPD and chronic bronchitis but no allergy
Source: Front Med (Lausanne). 2025 May 5;12:1572291. doi: 10.3389/fmed.2025.1572291 (PMC12087381; doi:10.3389/fmed.2025.1572291)

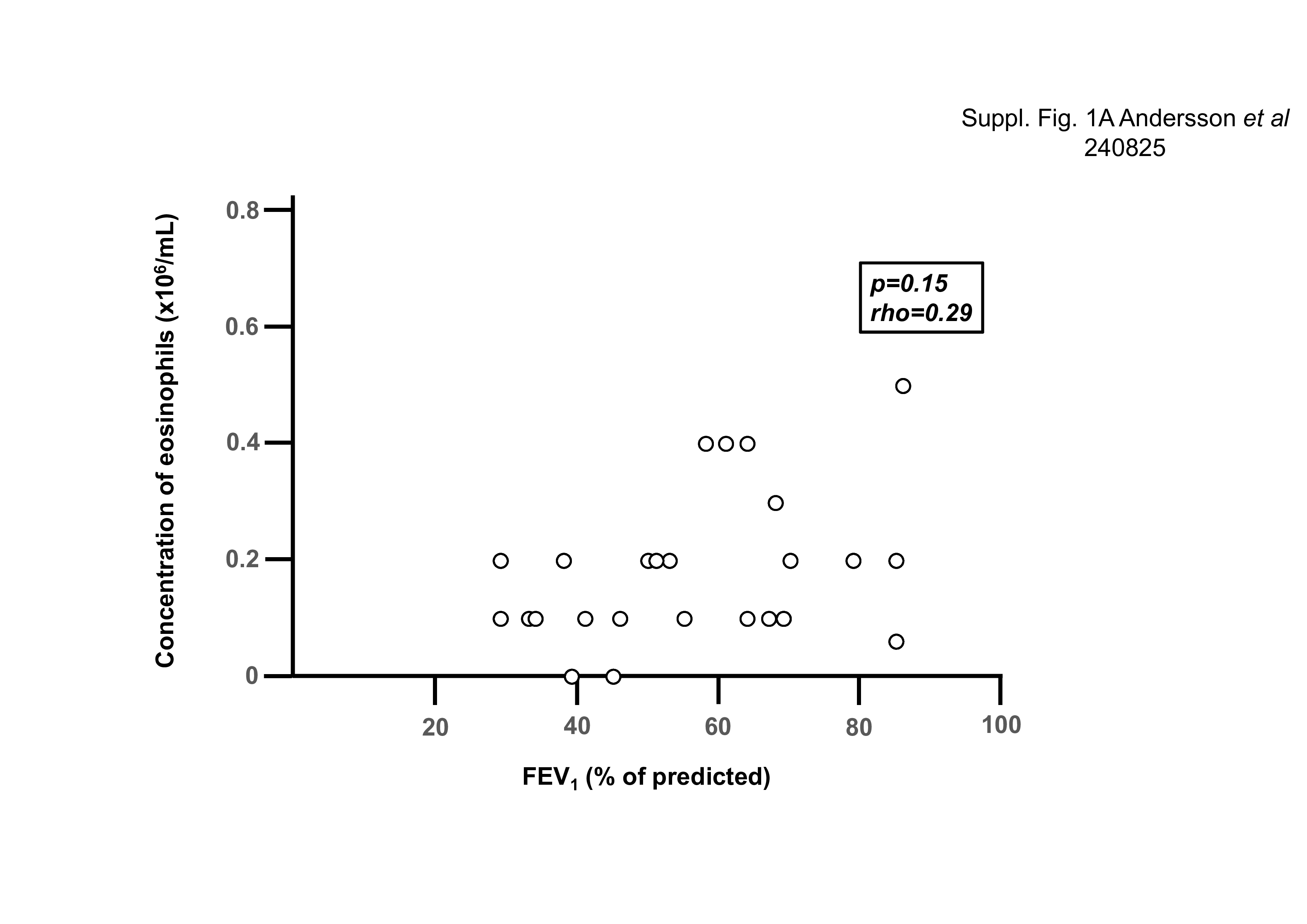

Supplement: Supplementary file 1 [file Image_1.TIFF]

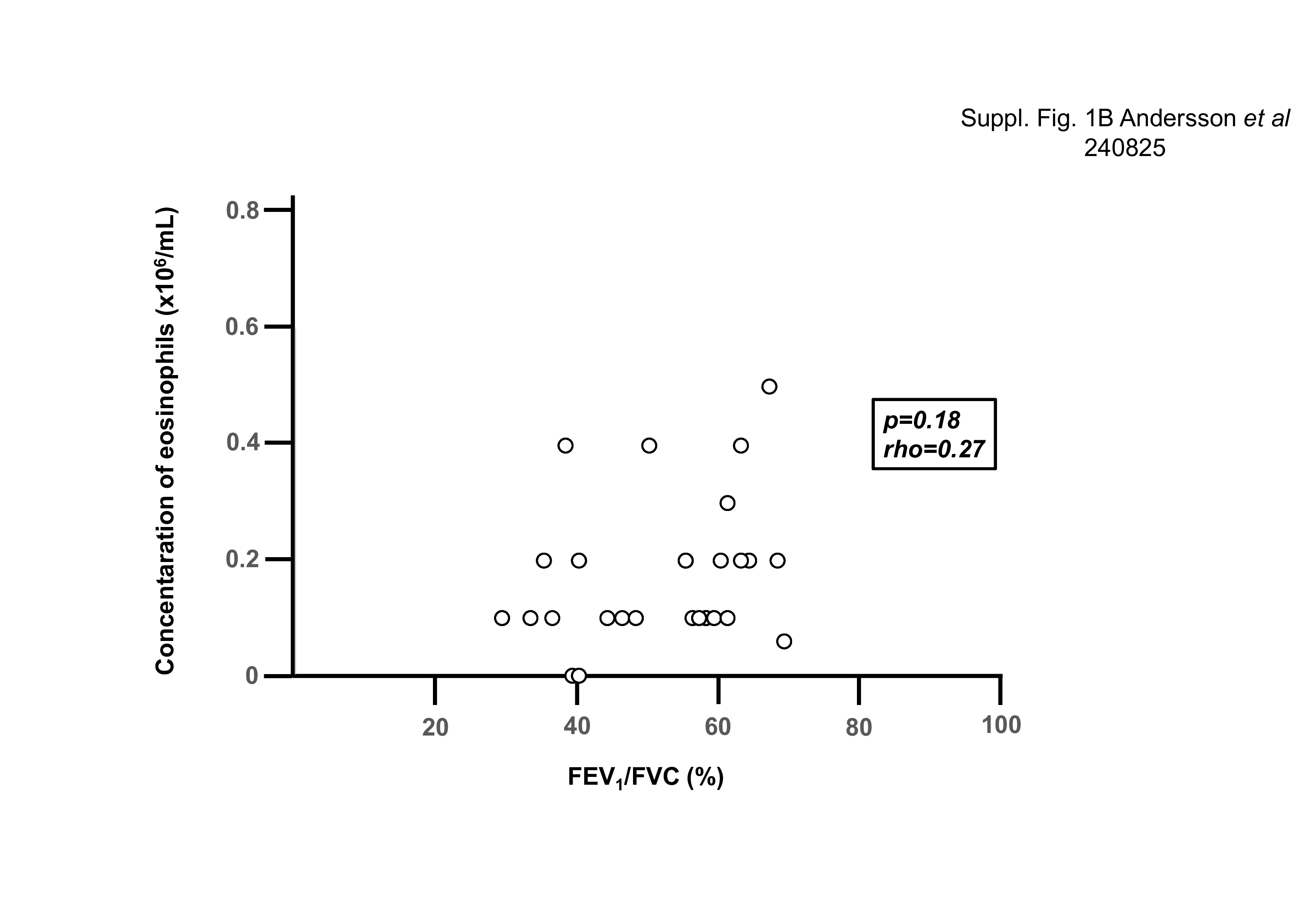

Supplement: Supplementary file 2 [file Image_2.TIFF]

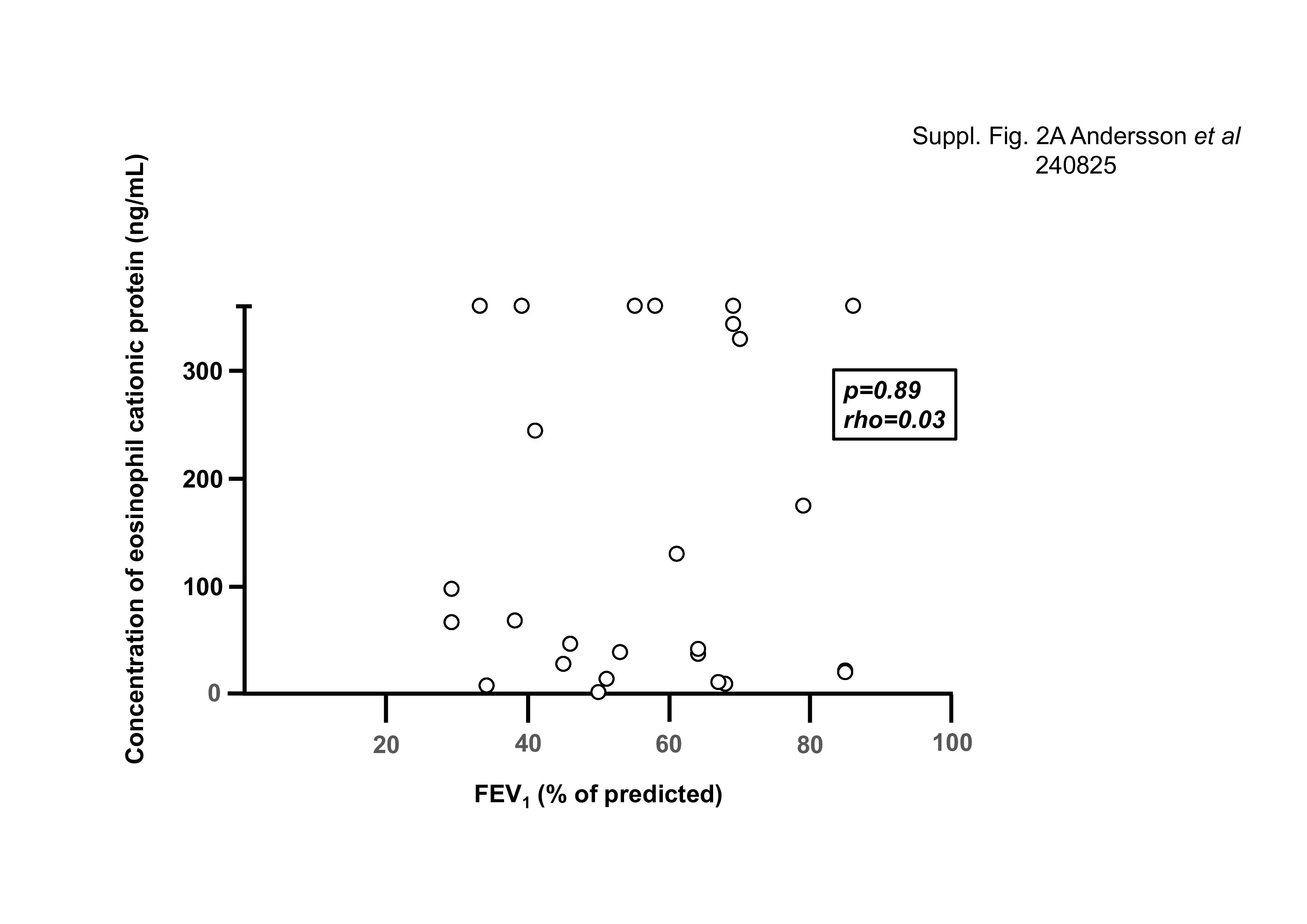

Supplement: Supplementary file 3 [file Image_3.TIFF]

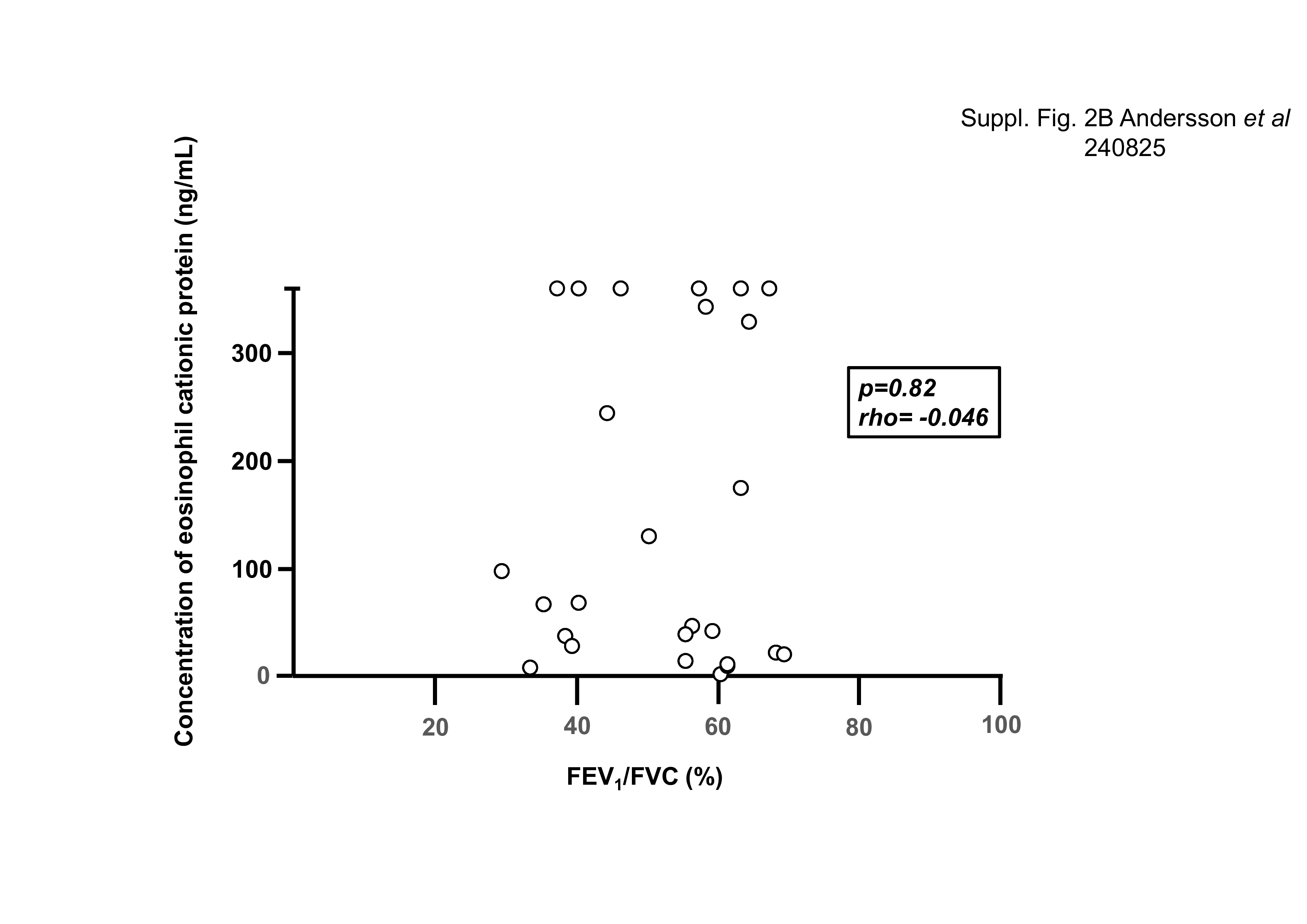

Supplement: Supplementary file 4 [file Image_4.TIFF]

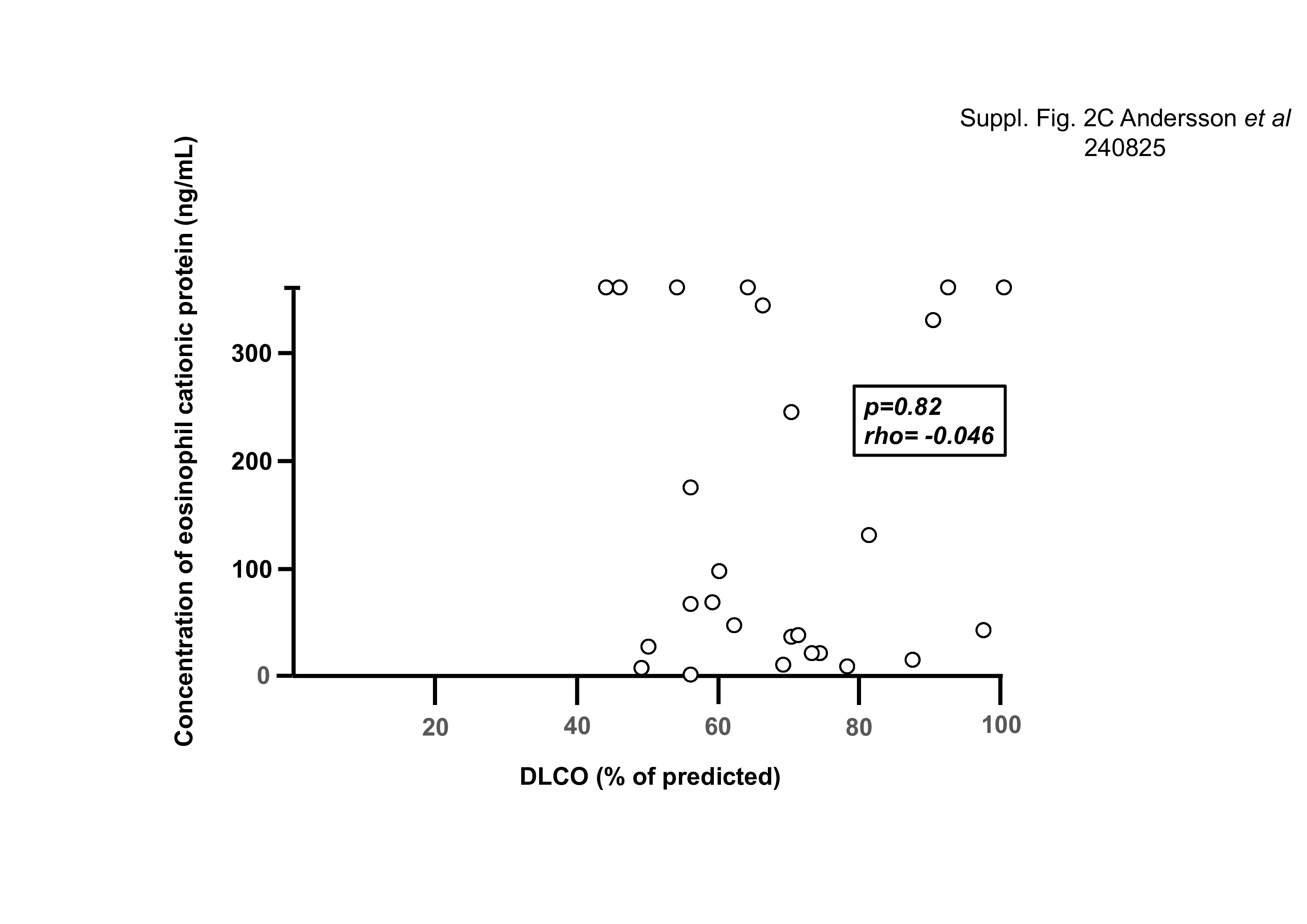

Supplement: Supplementary file 5 [file Image_5.TIFF]

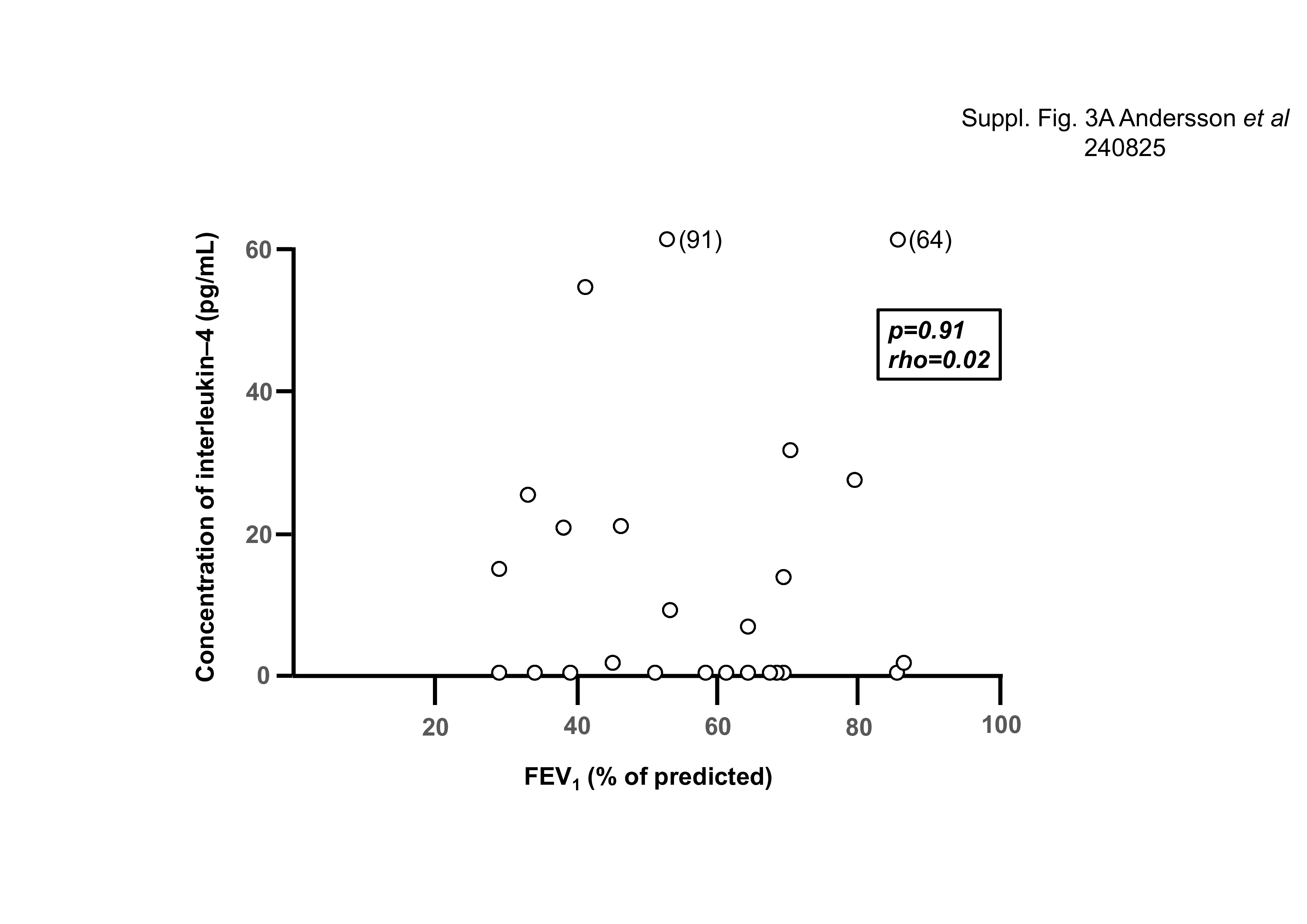

Supplement: Supplementary file 6 [file Image_6.TIFF]

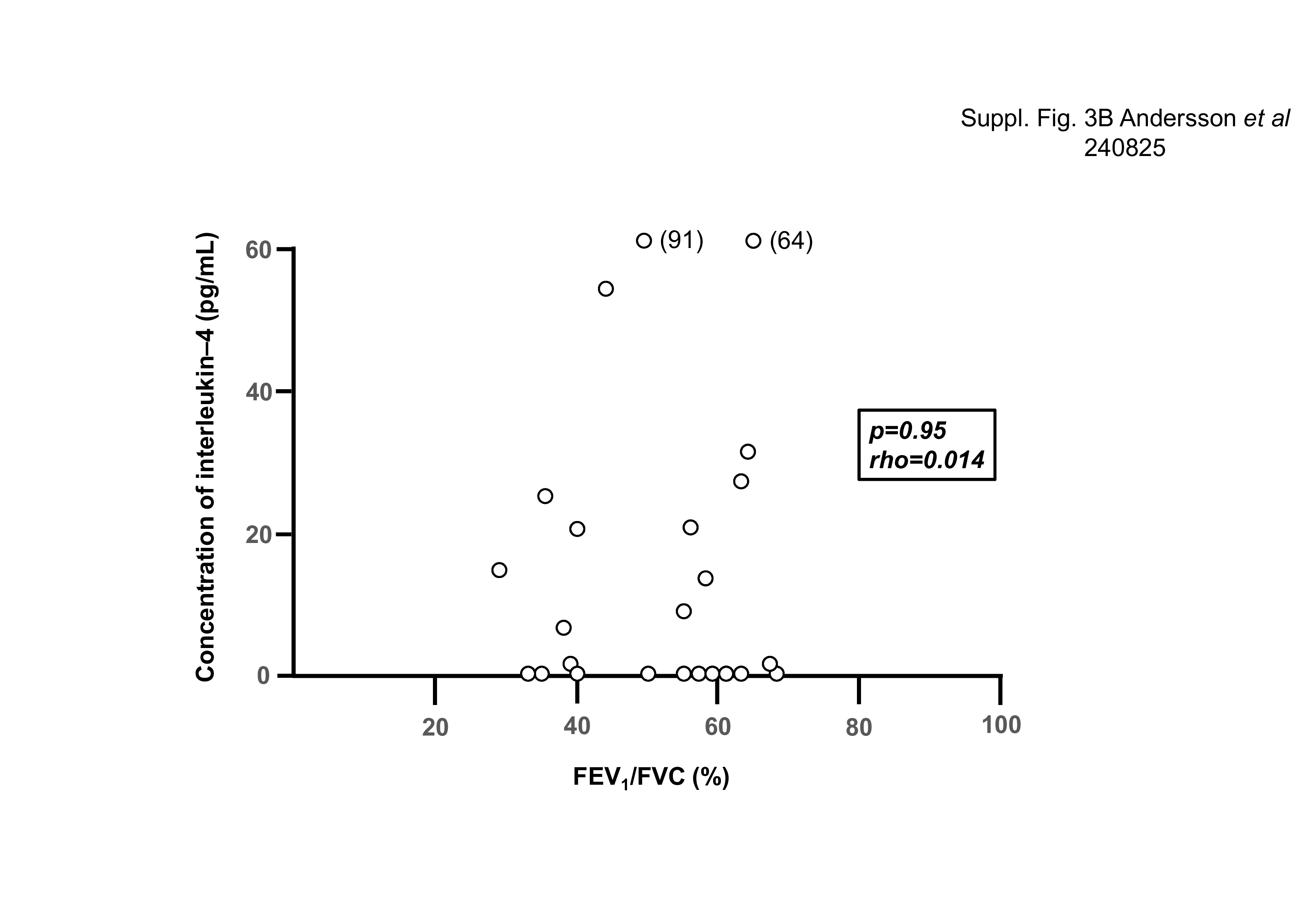

Supplement: Supplementary file 7 [file Image_7.TIFF]

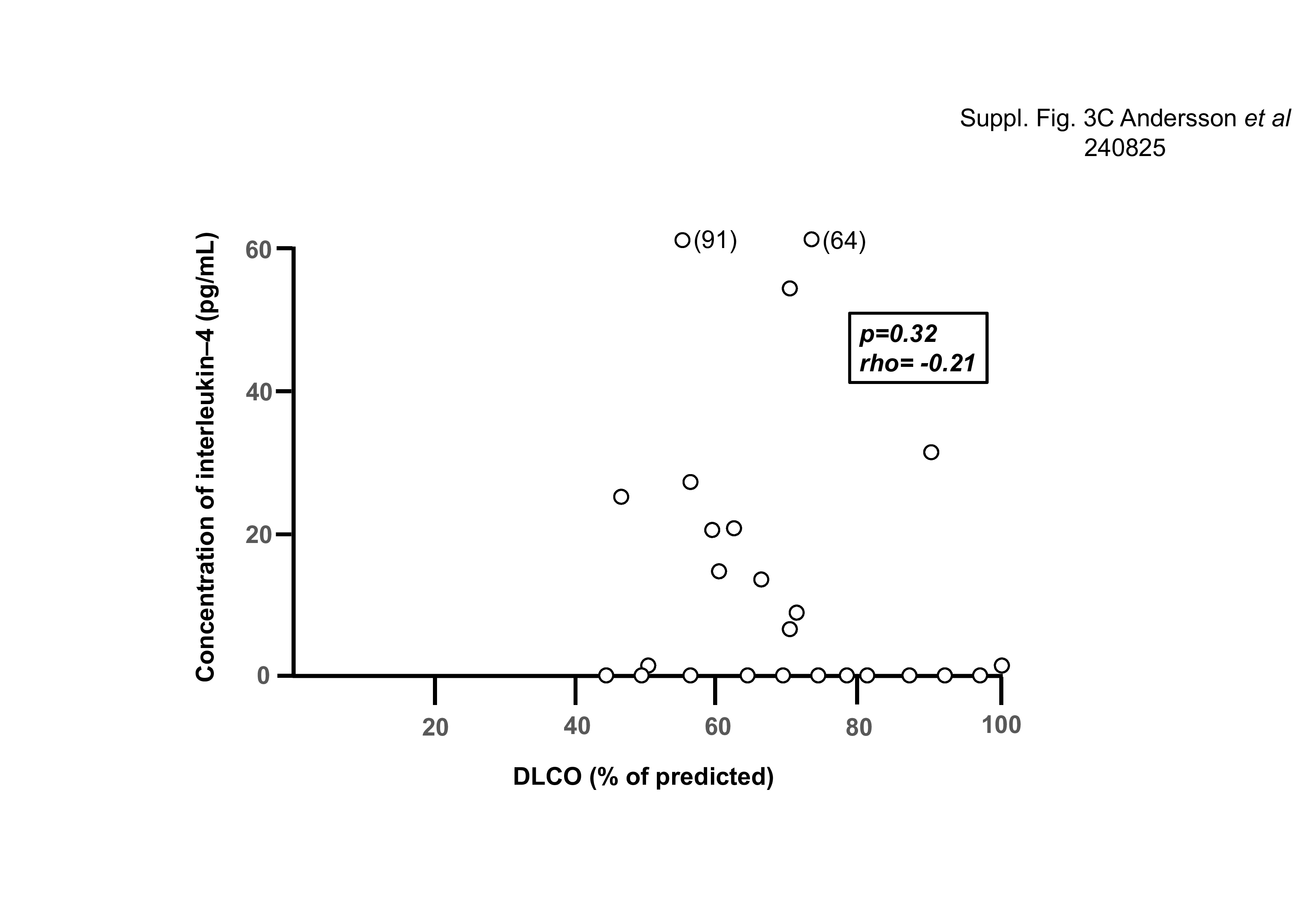

Supplement: Supplementary file 8 [file Image_8.TIFF]

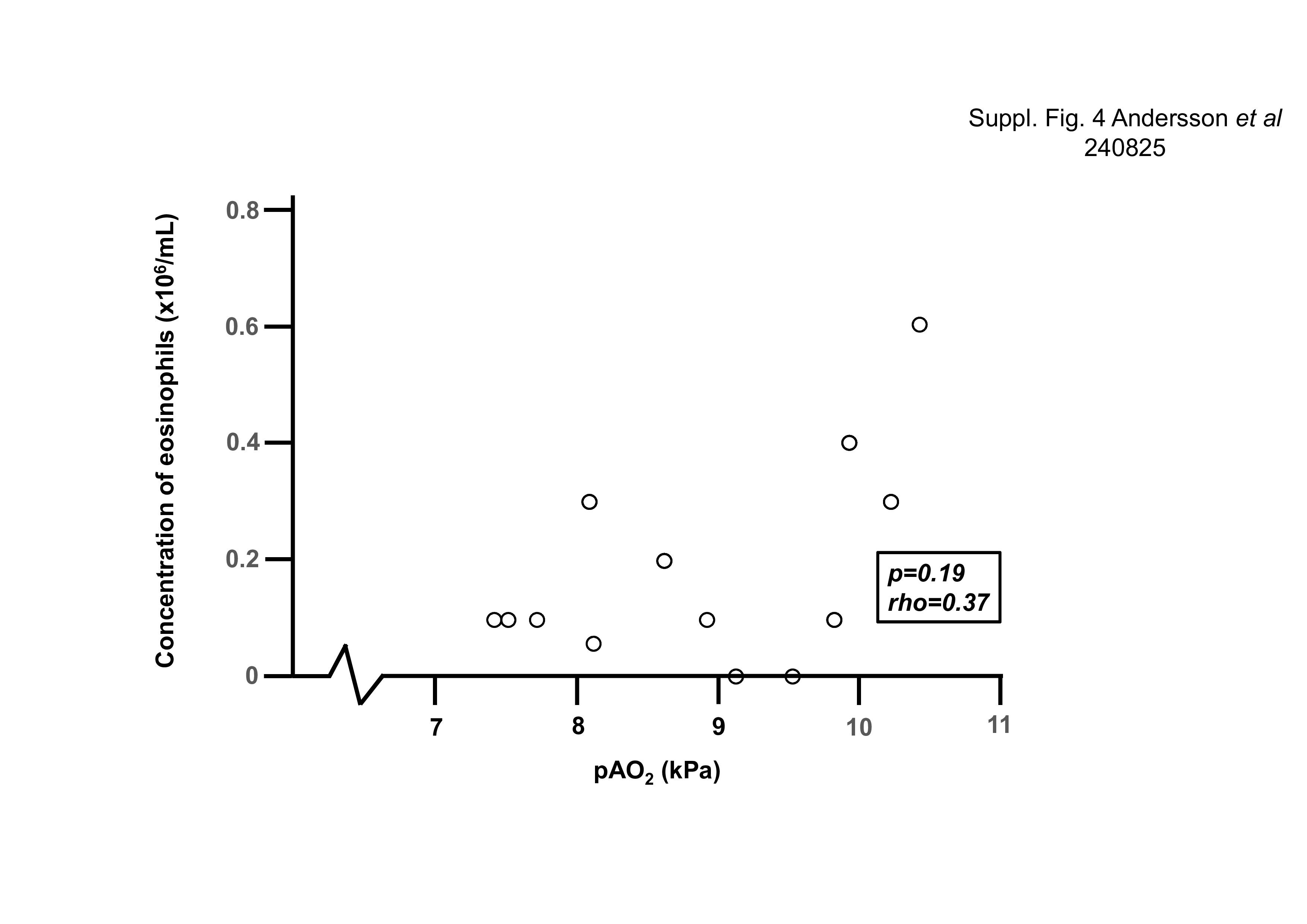

Supplement: Supplementary file 9 [file Image_9.TIFF]

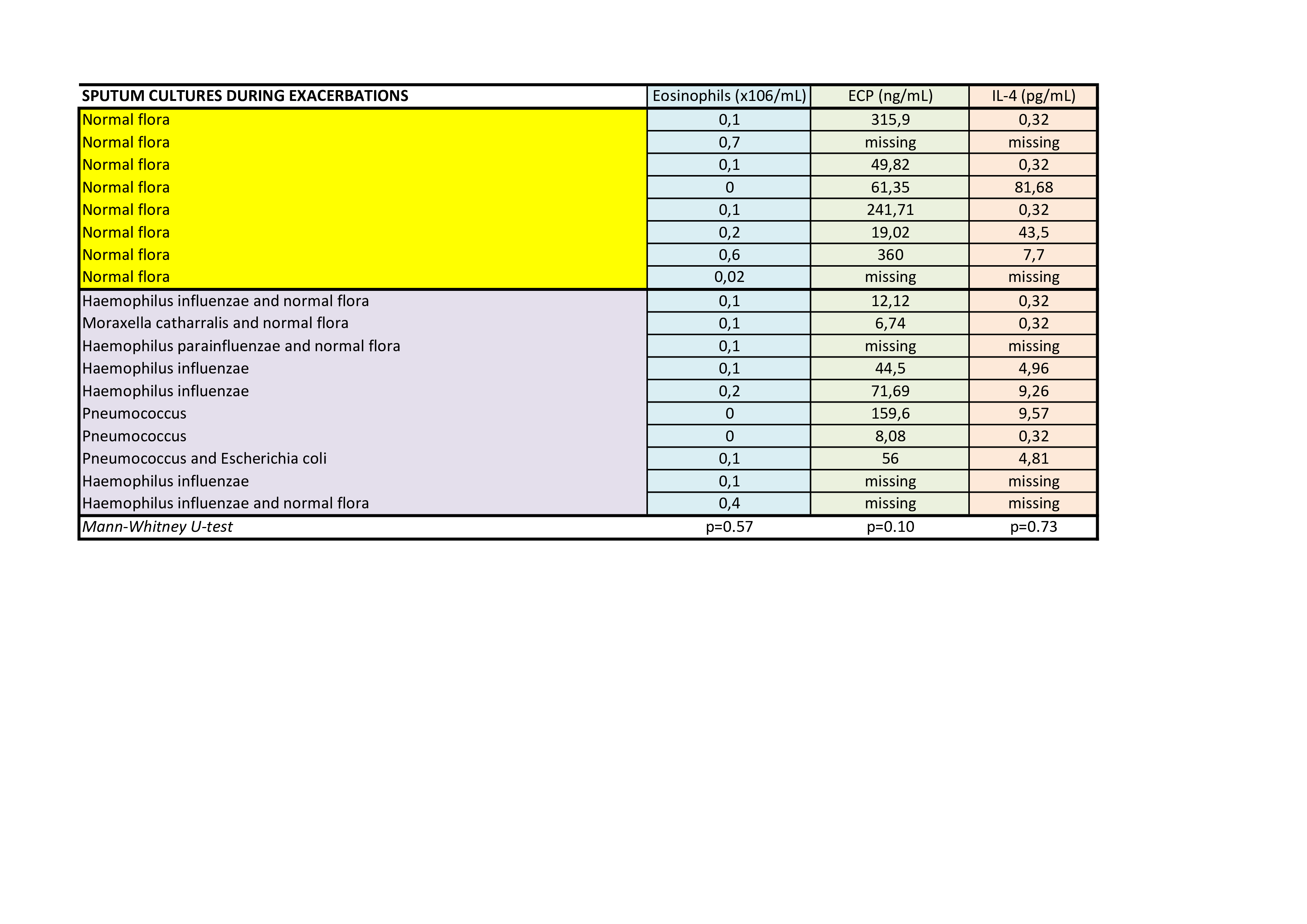

Supplement: Supplementary file 10 [file Image_10.TIFF]
